# The elderly age criterion for increased in-hospital mortality in trauma patients: a retrospective cohort study

**DOI:** 10.1186/s13049-021-00950-x

**Published:** 2021-09-10

**Authors:** Ji Hwan Lee, Min Joung Kim, Ju Young Hong, Jinwoo Myung, Yun Ho Roh, Sung Phil Chung

**Affiliations:** 1 Department of Emergency Medicine, Yonsei University College of Medicine, 50-1 Yonsei-ro, Seodaemun-gu, Seoul, 03722 Republic of Korea; 2 Biostatistics Collaboration Unit, Department of Biomedical Systems Informatics, Yonsei University College of Medicine, Seoul, Republic of Korea

**Keywords:** Trauma, Injury, Mortality, Elderly, Geriatric, Emergency Department-based Injury In-depth Surveillance

## Abstract

**Background:**

With an aging population, the number of elderly individuals exposed to traumatic injuries is increasing. The elderly age criterion for traumatic injuries has been inconsistent in the literature. This study aimed at specifying the elderly age criterion when the traumatic mortality rate increases.

**Methods:**

This is a multicenter retrospective cohort study that was conducted utilizing the data from the Emergency Department-based Injury In-depth Surveillance Registry of the Korea Disease Control and Prevention Agency, collected between January 2014 and December 2018 from 23 emergency departments. The outcome variable was in-hospital mortality. Multivariable logistic regression analysis was used to calculate the adjusted mortality rate for each age group. By using the shape-restricted regression splines method, the relationship between age and adjusted traumatic mortality was plotted and the point where the gradient of the graph had the greatest variation was calculated.

**Results:**

A total of 637,491 adult trauma patients were included. The number of in-hospital deaths was 6504 (1.0%). The age at which mortality increased the most was 65.06 years old. The adjusted odds ratio for the in-hospital mortality rate with age in the ≤ 64-year-old subgroup was 1.038 (95% confidence interval (CI) 1.032–1.044) and in the ≥ 65-year-old subgroup was 1.059 (95% CI 1.050–1.068). The adjusted odds ratio for in-hospital mortality in the ≥ 65-year-old compared to the ≤ 64-year-old subgroup was 4.585 (95% CI 4.158–5.055, *p* < 0.001).

**Conclusions:**

This study found that the in-hospital mortality rate rose with increasing age and that the increase was the most rapid from the age of 65 years. We propose to define the elderly age criterion for traumatic injuries as ≥ 65 years of age.

## Background

Worldwide, the aging population is rapidly increasing. Due to medical advances and public health measures, the number of elderly individuals working outdoors has been growing, which in turn increases the risk of traumatic injuries. In Korea, the number of individuals over 65 years of age who have died from traumatic injuries such as motor vehicle collisions (MVCs) and falls rose from 3280 to 2011 to 3742 in 2018 [1].

The physiological changes of aging cause various problems in the evaluation and treatment of trauma patients. The hospital to which the patient will be transferred is determined based on the field trauma triage guidelines, which encompass the mechanism of injury and low systolic blood pressure [2–5]. However, since these triage guidelines were developed using data containing the data from large number of young patients, it has been shown that the elderly are at risk of being under-triaged [6, 7]. Previous studies have reported that nearly half of the elderly patients with severe trauma were not transported to a suitable trauma center [8]. Moreover, the elderly trauma patient group had a higher rate of complications and in-hospital mortality than the younger patient group, however the activation rate of the trauma team was lower [9]. In addition, this age group has a high frequency of underlying comorbidities and medication use including anticoagulants, which are likely to adversely affect the prognosis. According to a study by Hollis et al. [10], 75.4% of trauma patients > 65 years of age have various underlying diseases, which was shown to be an independent predictor of mortality in these patients with an injury severity score (ISS) of ≤ 24 points.

Therefore, the medical staff should be able to clearly recognize elderly trauma patients who require more careful evaluation and treatment. However, the age criterion for defining the elderly in the field of trauma care is still unclear. The US Centers for Disease Control and Prevention recommends that trauma patients > 55 years of age be transferred to a trauma center, but according to the Vittel criteria, this recommendation applies to patients > 65 years [2, 5] illustrating the large gap between these guidelines. Like this, there is a big difference in the age criterion for the old age in the triage guidelines for trauma patients which is currently applied. In addition, the criterion for old age suggested in previous studies has been different, such as 56, 57, 60, 70 and 74 years of age [11–15]. This inconsistent age criterion has potential to confuse the medical staff and cannot be effectively applied in the clinical field. Therefore, there is a need to specify the age criterion in this subgroup of patients. This study aimed at specifying the elderly age criterion when the traumatic mortality rate increases.

## Methods

### Study design

This retrospective cohort study was conducted to evaluate the age criterion when the in-hospital mortality rate increases in elderly trauma patients. The data were collected through the Emergency Department-based Injury In-depth Surveillance (EDIIS) registry from 2014 to 2018, established by the Korea Disease Control and Prevention Agency. The EDIIS is a multi-center, prospective registry that collects data related to diseases or injuries caused by external factors such as trauma, poisoning, and environmental damage. This study was approved by Severance Hospital Institutional Review Board (Approval number: 4-2020-0707) and the need for informed consent was waived due to the nature of a retrospective study.

### Inclusion and exclusion criteria

This study included adult trauma patients older than 19 years of age who were treated in 23 emergency departments (ED) participating in the EDIIS between January 1, 2014, and December 31, 2018. The exclusion criteria were patients with non-traumatic injuries (poisoning, drowning, environmental damage, etc.), those who were dead on arrival, transferred from the ED to another hospital, and those with missing essential information (treatment results, trauma severity scale).

### Study data and variables

The variables collected from EDIIS included patient demographics (age, sex), vital signs upon ED arrival (blood pressure, heart rate, respiratory rate, body temperature), and Glasgow Coma Scale (GCS). As per the EDIIS data, trauma mechanisms were classified into MVCs, falls, blunt injuries, penetrating injuries, and other low-frequency mechanisms, such as injuries caused by explosions and foreign bodies. An Excessive Mortality Ratio-adjusted Injury Severity Score (EMR-ISS) was used as an index for evaluating the injury severity of the trauma, which is based on the patient diagnosis according to the 10th edition of the International Statistical Classification of Diseases and Related Health Problems [16]. The primary outcome was in-hospital mortality.

### Statistical analysis

Nominal variables were presented as frequencies and percentages and continuous variables that were not normally distributed were presented as median (inter quartile range, IQR). Statistical results were presented as odds ratio (OR) (95% confidence interval (CI)), and a *p*-value of < 0.05 was used for statistical significance. After adjusting for confounding variables, identified through logistic regression analysis, the shape-restricted regression splines method was used to evaluate the age criterion at which the mortality rate most rapidly increased [17] .This is a statistical technique that draws a nonlinear curve based on the distribution of predicated values calculated from a logistic regression model and evaluates the point where the slope of the curve has the largest variation. In order to secure the reliability of the calculated age criterion, using the bootstrapping method, random sample groups were repeatedly extracted 1,000 times from the study group, and the age cutoff was calculated from each sample. The mean value of the 1000 age cutoff calculated in this way and the 95% CI are presented together. In addition, age in the data was divided into groups of 10-year increments, and the average adjusted mortality rates were presented. Based on the calculated age cut-off point, the study group was divided into two, and subgroup analysis and comparison between them were performed. R package, version 3.4.3 (http://www.R-project.org) was used for the statistical analysis.

## Results

**Fig. 1:**
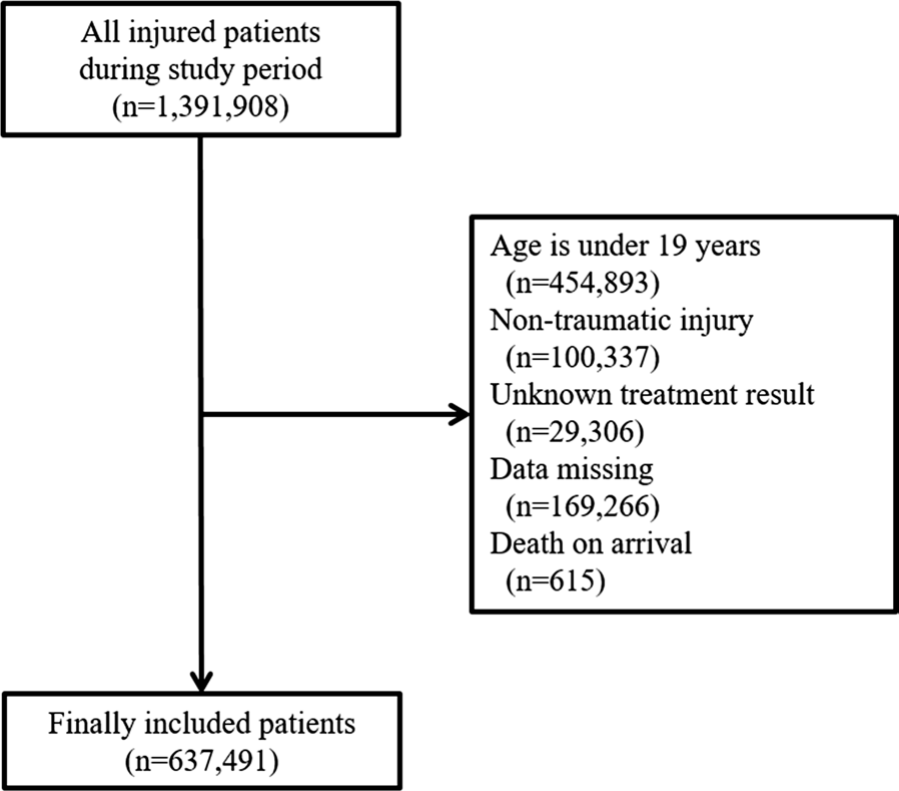
Flow diagram of the patients included in this study

During the study period, total 1,391,908 injured patients were treated in the ED which were participating in the EDIIS. Among them, 754,489 cases met the exclusion criteria, so finally the data of 637,491 trauma patients were included in this study. The median age of the patients was 47 years (32–61 years), and the proportion of women was 41.9%. The number of in-hospital deaths was 6504 (1.0%). The information of the process of collecting patients is presented in Fig. 1, and basic demographic information are presented in Table 1.

**Table 1:** Characteristics of the study population

Variables	Frequency (%) or median (IQR)
Age (years)	47 (32, 61)
*Sex*	
Female	267,281 (41.9)
Male	370,210 (58.1)
*Trauma mechanism*	
Motor vehicle collision	128,712 (20.2)
Fall	180,277 (28.3)
Blunt injury	129,734 (20.4)
Penetrating injury	41,810 (6.6)
Other^a^	156,958 (24.6)
*Vital signs*	
SBP (mmHg) (n = 530,933)	135 (120, 150)
DBP (mmHg) (n = 531,905)	80 (71, 90)
HR (beats/min) (n = 532,749)	81 (73, 91)
RR (breath/min) (n = 532,441)	20 (18, 20)
BT (℃) (n = 532,911)	36.5 (36.3, 36.8)
GCS (n = 133,587)	15 (15, 15)
EMR-ISS	9 (4, 11)
In-hospital mortality	6504 (1.0)

Total number of study population 637,491

BT, body temperature; CI, confidence interval; DBP, diastolic blood pressure; EMR-ISS, excess mortality ratio–adjusted Injury Severity Score; GCS, Glasgow Coma Scale; HR, heart rate; IQR, inter quartile range; OR, odds ratio; RR, respiratory rate; SBP, systolic blood pressure

^a^Other contains the low-frequency injury mechanisms, such as explosions or foreign bodies

In the univariable analysis, all variables were found to be associated with in-hospital mortality. However, in the multivariable analysis, body temperature did not show any statistical significance (Multivariable 1). Therefore, it was not included in the final regression model (Multivariable 2). An increase in age was found to be independently associated with an increase in in-hospital mortality (Adjusted OR (AOR) = 1.050, 95% CI 1.047–1.053, *p* < 0.001). In addition, sex, trauma mechanism, vital signs (excluding body temperature), GCS, and EMR-ISS were associated with in-hospital mortality. The logistic regression analysis results are presented in Table 2

**Table 2:** Univariable and multivariable logistic regression analyses of mortality

Variable	Univariable		Multivariable 1		Multivariable 2	
	OR (95% CI)	*p*-value	OR (95% CI)	*p*-value	OR (95% CI)	*p*-value
Age (years)	1.042 (1.040–1.043)	< 0.001	1.050 (1.047–1.053)	< 0.001	1.050 (1.047–1.053)	< 0.001
Female	0.616 (0.584–0.650)	< 0.001	0.787 (0.712–0.869)	< 0.001	0.791 (0.717–0.873)	< 0.001
*Trauma mechanism*						
Motor vehicle collision	Reference					
Fall	0.637 (0.603–0.674)	< 0.001	1.076 (0.962–1.202)	0.199	1.078 (0.965–1.204)	0.185
Blunt injury	0.103 (0.091–0.117)	< 0.001	0.457 (0.371–0.563)	< 0.001	0.462 (0.375–0.568)	< 0.001
Penetrating injury	0.154 (0.130–0.183)	< 0.001	0.678 (0.504–0.913)	0.499	0.676 (0.502–0.909)	0.010
Other^a^	0.294 (0.273–0.317)	< 0.001	0.631 (0.548–0.727)	< 0.001	0.629 (0.546–0.725)	< 0.001
*Vital signs*						
SBP (mmHg)	0.970 (0.969–0.970)	< 0.001	0.989 (0.987–0.990)	< 0.001	0.988 (0.987–0.990)	< 0.001
DBP (mmHg)	0.971 (0.970–0.972)	< 0.001	1.002 (1.001–1.003)	< 0.001	1.002 (1.001–1.003)	< 0.001
HR (beats/min)	0.989 (0.988–0.991)	< 0.001	1.003 (1.002–1.004)	< 0.001	1.003 (1.002–1.004)	< 0.001
RR (breath/min)	1.005 (1.005–1.005)	< 0.001	1.007 (1.005–1.008)	< 0.001	1.007 (1.006–1.008)	< 0.001
BT (℃)	1.003 (1.002–1.003)	< 0.001	1.000 (0.999–1.001)	0.737		
GCS	0.565 (0.561–0.570)	< 0.001	0.637 (0.630–0.644)	< 0.001	0.637 (0.630–0.644)	< 0.001
EMR-ISS	1.073 (1.072–1.074)	< 0.001	1.032 (1.030–1.035)	< 0.001	1.032 (1.030–1.035)	< 0.001

^a^Other contains the low-frequency injury mechanisms, such as explosions or foreign bodies

BT, body temperature; CI, confidence interval; DBP, diastolic blood pressure; EMR-ISS, excess mortality ratio–adjusted Injury Severity Score; GCS, Glasgow Coma Scale; HR, heart rate; OR, odds ratio; RR, respiratory rate; SBP, systolic blood pressure

### Age criterion for elderly trauma patients

**Fig. 2:**
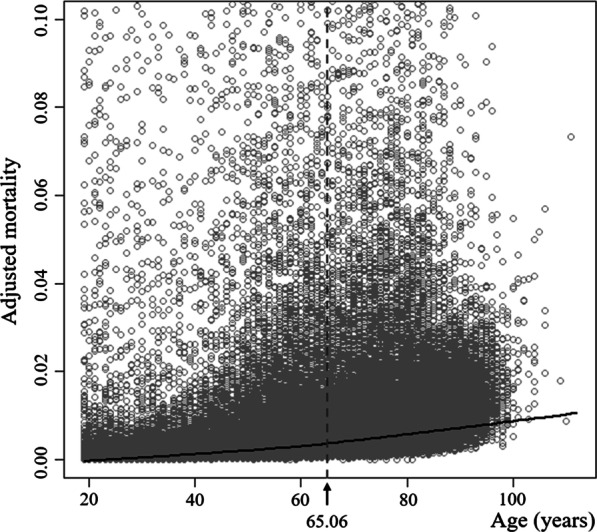
Results of the shape-restricted regression splines method of adjusted mortality rate

The distribution of predicated values and the nonlinear curve, calculated through the shape-restricted regression splines method, are presented in Fig. 2. There was a positive correlation between age and in-hospital mortality, and the point where the slope of the curve had the largest variation was at 65.06 years of age. The mean age criterion calculated by re-sampling 1000 times using the bootstrap technique was 64.74 years of age (95% CI 63.46–67.91).

**Fig. 3:**
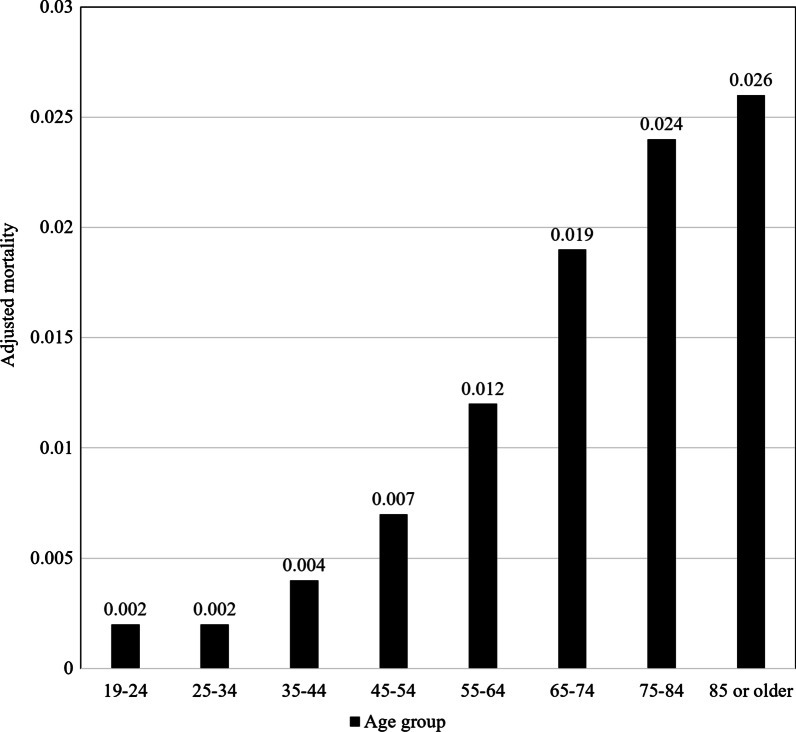
Average adjusted mortality rate for each age group of patients divided by 10-year increments

The average adjusted mortality rate for each group in the 10-year increments of age is presented in Fig. 3. The adjusted mortality rate for the 55–64-year-old group was 0.012, and the 65–74-year-old group was 0.019. The largest difference was found between these two groups.

The results of the multivariable logistic regression of subgroup analysis and intergroup comparison were presented in Table 3. The AOR for the in-hospital mortality rate with increasing age in the ≤ 64-year-old subgroup was 1.038 (95% CI 1.032–1.044, *p* < 0.001), and 1.058 (95% CI 1.049–1.067, *p* < 0.001) in the ≥ 65-year-old subgroup. The AOR for in-hospital mortality in the ≥ 65-year-old subgroup was 4.585 (95% CI 4.158–5.055, *p* < 0.001) compared to the ≤ 64-year-old subgroup.

**Table 3:** Multivariable logistic regression analysis of mortality by age subgroups

Variable	Subgroup analysis				Comparison between two groups	
	≤ 64 years of age		≥ 65 years of age		≥ 65 years of age^a^	
	OR (95% CI)	*p*-value	OR (95% CI)	*p*-value	OR (95% CI)	*p*-value
Age	1.038 (1.032, 1.044)	< 0.001	1.059 (1.050, 1.068)	< 0.001	4.585 (4.158, 5.055)	< 0.001
Female	0.723 (0.616, 0.850)	0.001	0.789 (0.696, 0.894)	< 0.001	0.813 (0.737, 0.897)	< 0.001
*Trauma mechanism*						
Motor vehicle collision			Reference			
Fall	1.290 (1.094, 1.522)	0.003	0.843 (0.725, 0.980)	0.026	1.224 (1.096, 1.366)	< 0.001
Blunt injury	0.463 (0.352, 0.610)	< 0.001	0.524 (0.377, 0.727)	< 0.001	0.462 (0.376, 0.568)	< 0.001
Penetrating injury	0.688 (0.464, 1.020)	0.064	0.701 (0.434, 1.132)	0.146	0.701 (0.523, 0.938)	0.017
Other^b^	0.617 (0.504, 0.756)	< 0.001	0.681 (0.560, 0.827)	< 0.001	0.686 (0.596, 0.789)	< 0.001
*Vital signs*						
SBP (mmHg)	0.986 (0.985, 0.988)	< 0.001	0.992 (0.990, 0.993)	< 0.001	0.989 (0.988, 0.990)	< 0.001
DBP (mmHg)	1.003 (1.002, 1.004)	< 0.001	1.002 (1.001, 1.003)	< 0.001	1.002 (1.002, 1.003)	< 0.001
HR (beats/min)	1.002 (1.001, 1.004)	0.011	1.003 (1.002, 1.005)	< 0.001	1.002 (1.001, 1.004)	< 0.001
RR (breath/min)	1.009 (1.007, 1.010)	< 0.001	1.004 (1.003, 1.005)	< 0.001	1.006 (1.005, 1.008)	< 0.001
BT (°C)						
GCS	0.603 (0.593, 0.613)	< 0.001	0.678 (0.668, 0.688)	< 0.001	0.635 (0.629, 0.642)	< 0.001
EMR-ISS	1.028 (1.025, 1.031)	< 0.001	1.036 (1.033, 1.039)	< 0.001	1.033 (1.031, 1.035)	< 0.001

BT, body temperature; CI, confidence interval; DBP, diastolic blood pressure; EMR-ISS, excess mortality ratio–adjusted Injury Severity Score; GCS, Glasgow Coma Scale; HR, heart rate; OR, odds ratio; RR, respiratory rate; SBP, systolic blood pressure

^a^Reference group is ≤ 64 years of age

^b^Other contains the low-frequency injury mechanisms, such as explosions or foreign bodies

### Variables influencing in-hospital trauma mortality

Female sex and higher systolic blood pressure were found to be associated with a lower mortality rate (AOR = 0.791, 95% CI 0.717–0.873, *p* < 0.001 and AOR = 0.988, 95% CI 0.987–0.990, *p* < 0.001, respectively). Increased heart and respiratory rates were associated with an increase in mortality. A higher GCS was associated with lower mortality rate (AOR = 0.637, 95% CI 0.630–0.644, *p* < 0.001), and a higher EMR-ISS was associated with higher mortality rate (AOR = 1.032, 95% CI 1.030–1.035, *p* < 0.001). These associations were seen in both subgroups of age. Regarding trauma mechanism, the mortality rate of falls was higher than that of MVCs in all patients and the ≤ 64-year-old subgroup, but in the ≥ 65-year-old subgroup, the mortality rate of MVCs was higher than falls (Table 3).

## Discussion

This study found an independent correlation between increasing age and increasing mortality in adult trauma patients; however, no obvious inflection point in the mortality rates between adjacent age group was noted. The statistically calculated elderly age criterion for increased in-hospital mortality from traumatic injuries was found to be 65 years of age. The AOR for in-hospital mortality was 4.585 (95% CI 4.158–5.055, *p* < 0.001) in the ≥ 65-year-old subgroup compared to the ≤ 64-year-old subgroup.

Previous studies have suggested the age criterion for elderly trauma patients, but each was conducted by selecting patients with different trauma severity. For example, Caterino et al. analyzed relatively severely injured trauma patients who died or were hospitalized for more than 48 h and found that the age criterion should be ≥ 70 years.[12] Kuhne et al. analyzed patients with an ISS score of ≥ 16 points and proposed that the age criterion should be ≥ 56 years, but Goodmanson et al. analyzed patients with minor traumatic injuries with an ISS score of ≤ 9 points and the proposed age criterion was ≥ 57 years [13, 15]. In Figs. 2 and 3, the difference in the trauma mortality rate between adjacent age groups was not large and tended to increase continuously. Based on this, the difference in the age criterion which was suggested in previous studies can be explained. If there is no obvious inflection point, there is a possibility that the age criterion was calculated differently based on the research method, such as the setting of the target population or studied variables in each study. Moreover, previous studies that included patients by the final trauma evaluation are limited in that their results cannot be applied to the field triage stage where such information cannot be readily obtained. Therefore, in order to establish the age criterion for the elderly that can be used for triage, it is necessary to conduct studies based on all trauma patients, not the selected trauma patient group, as in the present study.

Similar to our study, Campbell et al. analyzed the in-hospital mortality rate of all adult trauma patients, and they suggested that the age criterion should be ≥ 60 years [11]. In the graph of association between age and trauma mortality presented in their study, the mortality slope increased at the age of 37 and 60, and the slope decreased at the age of ≥ 78 years. Finally, the graph was in the form of a sigmoid function. Similarly, in our study, Fig. 3 shows that the mortality slope began to increase from the 35–44-year-old group, and the slope decreased from the 85-year-old group. This type of graph suggests that the age criterion for the largest increase in trauma mortality is at the position excluding both extremes of the adult age group. Therefore, if data is accumulated through subsequent studies, it is expected that a more sophisticated age criterion for the elderly can be established that can be used in the clinical setting.

Sex, trauma mechanism, vital signs (except body temperature), GCS, and EMR-ISS were found to be associated with in-hospital mortality, and these associations were present in both age subgroups divided by the age criterion. However, regarding the trauma mechanism, the mortality rate of MVCs was higher than that of falls in the total study population and the ≥ 65-year-old subgroup, but in the ≤ 64-year-old subgroup, the mortality of falls was higher than MVCs. This difference is assumed to be due to the different rates of high energy fall between the two subgroups. In fact, the proportion of falls from stairs or a height of ≥ 1 m, considered as high energy injury, was 24.19% in the ≤ 64-year-old group and 14.97% in the ≥ 65-year-old group.

There was no obvious inflection point in the mortality slope in this study. This indicates that identification of elderly patients by the age criterion alone is limited. Research aimed at establishing the age criterion for elderly patients, such as the presented study, assumes that patients who have same age possess the same frailty. However, even in patients of the same age, there is a difference in the health status, such as the degree of independent activities or underlying diseases; therefore, there will be a difference in frailty. Recent studies reported that that the degree of frailty, which is measured by an easily accessible tool for frailty is related to trauma mortality [18, 19]. Therefore, we expect that more meaningful results would be obtained if studies are conducted to establish the definition of elderly reflecting the degree of frailty along with age.

There are several limitations to this study. First, the study data were collected from a single country. Therefore, the result cannot be generalized to other countries. Second, due to the multi-center registry, there could be a selection bias that occurs due to differences in treatment strategies of each institution, as well as some missing data [20]. Third, all institutions participating in the EDIIS are referral medical institutions, thus they could have a higher proportion of severely injured patients compared to all trauma patients. Fourth, there are several variables known to be associated with trauma mortality, for example, underlying disease, medication history, and degree of frailty, which were not analyzed in this study. Fifth, the age criterion suggested in this study was calculated by a statistical method; however, there was no obvious inflection point detected in the mortality graph. Lastly, since the outcome variable was limited to in-hospital mortality, deaths before arrival or after discharge were not included. There is a possibility that the age criterion could change if these deaths were accounted for in the analysis.

## Conclusions

This study found that the in-hospital mortality rate rose with increasing age and that the increase was the greatest from the age of 65 years. The ≥ 65-year-old subgroup was approximately 4.6 times more likely to die in-hospital from traumatic injuries than the ≤ 64-year-old subgroup. Based on these results, we propose to define the elderly age criterion for traumatic injuries as ≥ 65 years of age. We hope that further studies are conducted to accurately define elderly patients.

## Data Availability

The Korea Disease Control and Prevention Agency prohibits the disclosure of research data collected through EDIIS without permission. The data that support the findings of this study are available from the Korea Disease Control and Prevention Agency but restrictions apply to the availability of these data, which were used under license for the current study, and so are not publicly available. Data are however available from the authors upon reasonable request and with permission from the Korea Disease Control and Prevention Agency.

## Abbreviations

AOR: Adjusted odds ratio
CI: Confidence interval
ED: Emergency department
EDIIS: Emergency Department-based Injury In-depth Surveillance
EMR-ISS: Excessive Mortality Ratio-adjusted Injury Severity Score
GCS: Glasgow Coma Scale
ISS: Injury severity score
MVC: Motor vehicle collision
OR: Odds ratio
